# Effect of Acclimatization in Elevated CO_2_ on Growth and Aflatoxin B_1_ Production by *Aspergillus flavus* Strains on Pistachio Nuts

**DOI:** 10.3390/microorganisms10010049

**Published:** 2021-12-27

**Authors:** Alaa Baazeem, Angel Medina, Naresh Magan

**Affiliations:** 1 Department of Biology, College of Science, Taif University, P.O. Box 11099, Taif 21944, Saudi Arabia; aabaazeem@tu.edu.sa; 2 Applied Mycology Group, School of Water, Energy and Environment, Cranfield University, Cranfield MK43 0AL, UK; a.medinavaya@cranfield.ac.uk

**Keywords:** *Aspergillus*, aflatoxin B_1_, climate change, acclimatization, growth, resilience, interacting abiotic factors, drought stress

## Abstract

There is little knowledge of the effect of acclimatization of *Aspergillus flavus* strains to climate-related abiotic factors and the subsequent effects on growth and aflatoxin B_1_ (AFB_1_) production. In this study, two strains of *A. flavus* (AB3, AB10) were acclimatized for five generations in elevated CO_2_ (1000 ppm × 37 °C) on a milled pistachio-based medium. A comparison was made of the effects of non-acclimatized strains and those that were acclimatized when colonizing layers of pistachio nuts exposed to 35 or 37 °C, 400 or 1000 ppm CO_2_, and 0.93 or 0.98 water activity (a_w_), respectively. Acclimatization influenced the fitness in terms of the growth of one strain, while there was no significant effect on the other strain when colonizing pistachio nuts. AFB_1_, production was significantly stimulated after ten days colonization when comparing the non-acclimatized and the acclimatized AB3 strain. However, there was no significant increase when comparing these for strain AB10. This suggests that there may be inter-strain differences in the effects of acclimatization and this could have a differential influence on the mycotoxin contamination of such commodities.

## 1. Introduction

Global warming has received significant interest because of the concerns regarding its impact on food security, quality, and safety. The challenge of increasing crop productivity to meet the demand of an ever-growing world population may be further exacerbated by climate change (CC) scenarios [1]. Under CC conditions, plant growth and physiology will be modified together with the exposure to different fungal pathogens and pests [2,3]. This has been shown to also change the regions where suitable environmental conditions occur for fungal infection and mycotoxin contamination [4,5]. Indeed, it has been shown that the colonization of maize by *Aspergillus flavus* under CC-related abiotic factors results in the stimulation of aflatoxin B_1_ (AFB_1_) contamination [6,7]. Recently, Baazeem et al. [8] showed that strains of *A. flavus* isolated from pistachio nuts grew optimally at around 35 °C on both pistachio nut-based media and on raw pistachio nuts. In contrast, AFB_1_ production was optimum at around 30 °C. The impact of CC conditions of elevated temperature (35 vs. 37 °C) and CO_2_ (400 vs. 1000 ppm) under conducive and stressed water availability conditions had little effect on the growth of strains of *A. flavus*. However, there were indications of a stimulation of AFB_1_ production, both *in vitro* and on raw pistachio nuts [9]. Indeed, there were also changes in the expression of some of the structural and regulatory genes (*aflD* and *aflR*) involved in toxin biosynthesis. Some strain variabilities were also observed.

There has been interest in the resilience of fungal pathogens, including mycotoxigenic species, when repeatedly exposed to CC interacting abiotic conditions. This is usually referred to as “acclimatization”. This is defined as a process in which an individual organism adjusts to a change in its environment, allowing it to maintain fitness. This may be important, especially if pathogenicity/virulence is modified or if mycotoxin production is increased. Vary et al. [10] carried out one of the first studies that examined the acclimatization of fungal pathogens of wheat in relation to CC abiotic factors. They found that wheat plants exposed to 350 vs. 650 ppm CO_2_ in controlled plant growth chambers had effects on crop physiology and severity of wheat diseases. Thus, ripening wheat ears exposed to CC conditions changed the stomatal number, position, and physiology of the plant. *Septoria tritici* and *Fusarium graminearum* sub-cultured for 10 and 20 generations in elevated CO_2_ conditions, respectively, were found to change their infection and disease symptoms after such acclimatization. Thus, the acclimatized strains of the two fungal pathogens had increased pathogenicity under CC conditions, with both disease symptoms and fungal biomass using molecular approaches shown to increase. While there was an increase in *Fusarium* Head Blight symptoms, no quantification was made of whether concomitant contamination with type B trichothecenes also occurred. Recently, Mshelia et al. [11] acclimatized one strain each of *F. verticillioides* and *F. graminearum* for 10 generations on a milled maize-based media. However, this was only in relation to temperature, with exposure to step-wise increases in 0.5 °C steps for each generation, from 30 to 35 °C for the former, and from 30 to 33 °C for the latter species. No acclimatization by exposing to elevated CO_2_ were included. Subsequent exposure of these pathogens to changes in temperature × elevated CO_2_ (400 vs. 800, and 1200 ppm) × water activity (0.92, 0.95, and 0.98 a_w_) showed little effect on growth or fumonisins, and deoxynivalenol or zearalenone production by *F. verticilioides* and *F. graminearum,* respectively.

Pistachio nuts are known to be more prone to contamination with AFB_1_ when there is increased insect infection and poor post-harvest practices, which could, under CC scenarios, predispose these tree nuts to *A. flavus* infection and perhaps increased AFB_1_ contamination. The evolution of acclimatization to facilitate better resilience to climate change scenario abiotic conditions and thus ecological fitness, by perhaps increasing toxin production, has not been examined previously in any food raw commodities. Indeed, to our knowledge, there have been no studies on the impact that the acclimatization of *A. flavus* strains to elevated CO_2_ may have on the colonization and AFB_1_ contamination of tree nuts.

The objectives of the present study were to examine whether acclimatization to 1000 ppm elevated CO_2_ + 37 °C of *A. flavus* strains (AB3 and AB10) for five generations would affect (a) mycelial colonization and (b) AFB_1_ contamination of raw pistachio nuts under interacting CC conditions (35 vs. 37 °C, 0.98 or 0.93 a_w_ × 400 vs. 1000 ppm CO_2_), and to compare this with non-acclimatized strains under existing and future environmental conditions.

## 2. Materials and Methods

### 2.1. Apergillus flavus Strains

Two strains of *A. flavus* were selected for this study due to their ecophysiological similarities to the type strain based on preliminary experiments. These strains were AB3 and AB10 [7].

### 2.2. Acclimatization Experimental System

Both *A. flavus* strains were acclimatized by exposure to 1000 ppm elevated CO_2_ at 37 °C with repeated sub-culturing for five generations. Each generation was incubated for 7–10 days on a 2% milled pistachio nut-based medium in a climate change growth chamber. For each generation, the mycelial hyphal tips (2.5 mm agar blocks) from the edge of the growing colony were carefully cut with a surface-sterilized scalpel blade and centrally placed on a fresh pistachio nut-based medium to initiate the next generation. This process was repeated for five generations.

### 2.3. Colonisation of Pistachio Nuts by Aspergillus flavus Isolates and Incubation Conditions

For experiments with non-acclimatized and acclimatized strains, the a_w_ of the raw pistachio nuts was modified to 0.98 and 0.93 a_w_ by adding known amounts of water based on the previously developed moisture sorption curves, and they were left overnight at 4 °C for equilibration [7]. A conidial spore suspension from non-acclimatized and acclimatized strains (0.2 mL; 10^6^ conidia/mL) was spread-plated using a bent surface sterilized glass rod on to 2% milled pistachio nut medium in 9 cm Petri pates. These were left overnight at 30 °C to allow initial germination to occur. Using a surface-sterilised 4 mm cork-borer and needle a single agar disc containing the conidial germlings was carefully placed in the centre of the layers of raw pistachio nuts in each replicate Petri plate for the different treatment a_w_ levels [8]. They were incubated under existing conditions: 35 °C + 400 ppm CO_2_ + 0.98 or 0.93 a_w_, and CC conditions of 37 °C + 1000 ppm CO_2_ + 0.98 or 0.93 a_w_.

All of the experiments were carried out in environmental growth chambers, as described previously [8]. Briefly, inoculated replicates and treatments were placed in plastic environmental chambers. Inoculated treatments of the same a_w_ were enclosed together in the environmental chambers containing switchable valves at each end: one for CO_2_ intake and the other for exit. Two 500-mL beakers of glycerol/water solution with the same a_w_ as the treatment were included in the chamber in order to maintain the same equilibrium relative humidity (ERH) as the media a_w_.

Colonization of the raw pistachio nut layers in 9 cm Petri plates was measured every two days. Immediately after measurement, the environmental chambers were flushed with the treatment CO_2_ for 10–15 min and then sealed and incubated at the treatment temperature. The chambers were flushed every 24 h with either 5 L of air (400 ppm CO_2_) or 1000 ppm CO_2_ from a specialty gas cylinder (British Oxygen Company Ltd., Guilford, Surrey, UK; 1000 ppm CO_2_ cylinder). They were incubated for 10 days at 35 and 37 °C.

At the end of the 10 days, the pistachio nuts were dried at 50 °C and milled for AFB_1_ extraction and quantification.

### 2.4. Quantification of Aflatoxin B_1_ Production

The dried pistachio nut samples were ground (Waring blender, Merck Ltd., Feltham, UK) and weighed (25 g). The background aflatoxin B_1_ levels in the nuts used in the experiments was 0.015 ng/g. This was taken into account as a correction factor in the final quantification of the results. Acetonitrile/water 60/40 (100 mL) was used as an extraction solvent. The mixture was blended for 3 min and the extract filtered into a smaller sample container. A PBS buffer (pH 7.4, Thermo Fisher Scientific, Waltham, MA, USA) was used for sample dilution, then the diluted extract was passed through an Immunoaffinity Column (IAC; AflaStar™; Romer Labs, Tulln, Austria) with a flow rate between 1–3 mL/min. The column was rinsed with 2 × 10 mL sterile distilled water. HPLC-grade methanol (1.5–3 mL) was then applied to the column and the eluent was collected in a new amber glass vial and left to dry overnight at room temperature before derivatisation.

Derivatisation of Aflatoxin B_1_ extract: Derivatisation of the AFB_1_ extract was performed according to the AOC method [12]. First, 200 μL hexane was added to the tube, followed by 50 μL of triflouroacetic acid. The mixture was vortexed for 30 s and left for 5 min. A mixture of water/acetonitrile (9:1) was then added to the tube, and vortexed for 30 s and left for 10 min to allow for separation of the layers. Then, the aqueous layer was filtered using a nylon syringe filter (13 mm × 0.22 μm; Jaytee Biosciences Ltd., Herne Bay, UK) into amber salinized 2 mL HPLC vials (Agilent, Santa Clara, CA, USA) before the HPLC analysis. All of the analytical reagents used were HPLC-grade.

Quantification of aflatoxin B_1_ with High Performance Liquid Chromatography HPLC: A reverse-phase HPLC with fluorescence detection was used to confirm the identity and quantify AFB_1_. An Agilent 1200 series HPLC system was used for the analysis. It consisted of an in-line degasser, auto sampler, binary pump, and a fluorescence detector (excitation and emission wavelengths of 360 and 440 nm, respectively). Separation was achieved using a C18 column (Phenomenex Gemini; 150 × 4.6, 3 μm particle size; Phenomenex, Torrance, CA, USA) with a Phenomenex Gemini C18 3 mm, 3 μm guard cartridge. Isocratic elution with methanol/water/acetonitrile (30:60:10, *v*/*v*/*v*) as the mobile phase was performed at a flow rate of 1.0 mL/min. The injection volume was 20 µL. A set of standards was injected (1 to 5 ng AFB_1_, AFB_2_, AFG_1_, and AFG_2_ per injection) and standard curves were generated by plotting the area underneath the peaks against the amounts of AFB_1_ standard injected.

### 2.5. Statistical Analysis

Three replicates per treatment were used in all of the experimental studies. The means were obtained by taking the average of each three measurements with the standard error of the means (±SE). Analysis of variance (ANOVA) was applied to analyze the variation of means with a 95% confidence interval. Normal distribution of data were checked with the normality test of Kolmogorov−Smirnov using Minitab statistical software. Fisher’s least significant difference (LSD) was used to identify the differences between the means, with *p* < 0.05 as a significant difference using the same statistical software.

## 3. Results

### 3.1. In Situ Effects of Acclimatization on Colonisation of Pistachio Nuts by A. flavus Strains under Existing and Climate-Related Abiotic Factors

**Figure 1:**
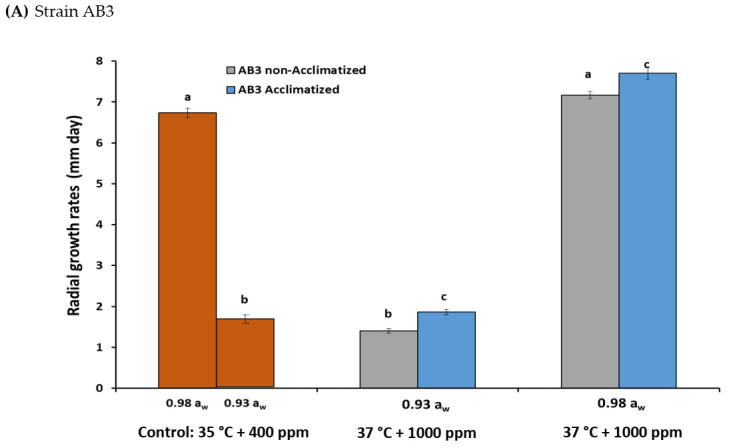

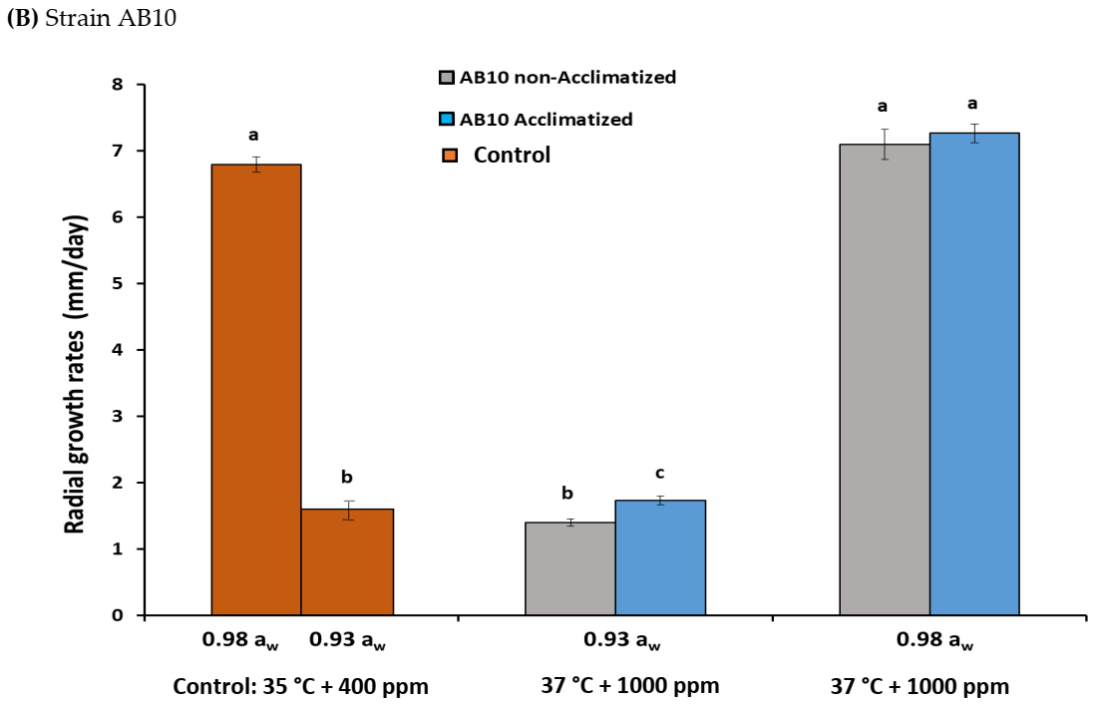
(**A**,**B**) Comparison of the radial growth rates (mm/day) of non-acclimatised and acclimatised (for five generations) strains of *Aspergillus flavus* strains (AB3 and AB10) when colonising layers of raw pistachio nuts incubated in control conditions (35 °C + 400 ppm CO_2_) and at 37 °C + 1000 ppm CO_2_ at 0.98 and 0.93 water activity (a_w_). Data are the means of triplicates. Bars represent SEM. Different letters indicate significant differences between treatments (*p* < 0.05) using Fisher’s least significant difference (LSD).

Figure 1A,B shows the effects of acclimatization on growth of two strains of *A. flavus* (AB3 and AB10) under interacting climate change-related abiotic factors. The colonisation of raw pistachio nuts of strain AB3, which were acclimatized for five generations and then exposed to climate change conditions, was changed, at both 0.93 and 0.98 a_w_. For strain AB10, the original strain as well as the one acclimatized (for five generations) showed little difference in the colonization rates of raw pistachio nuts, except at 0.93 a_w_, under the interacting abiotic conditions.

### 3.2. In Situ Effects of Acclimatization of A. flavus Strains on Aflatoxin B_1_ Contamination of Raw Pistachio Nuts under Existing and Climate-Related Abiotic Factors

**Figure 2:**
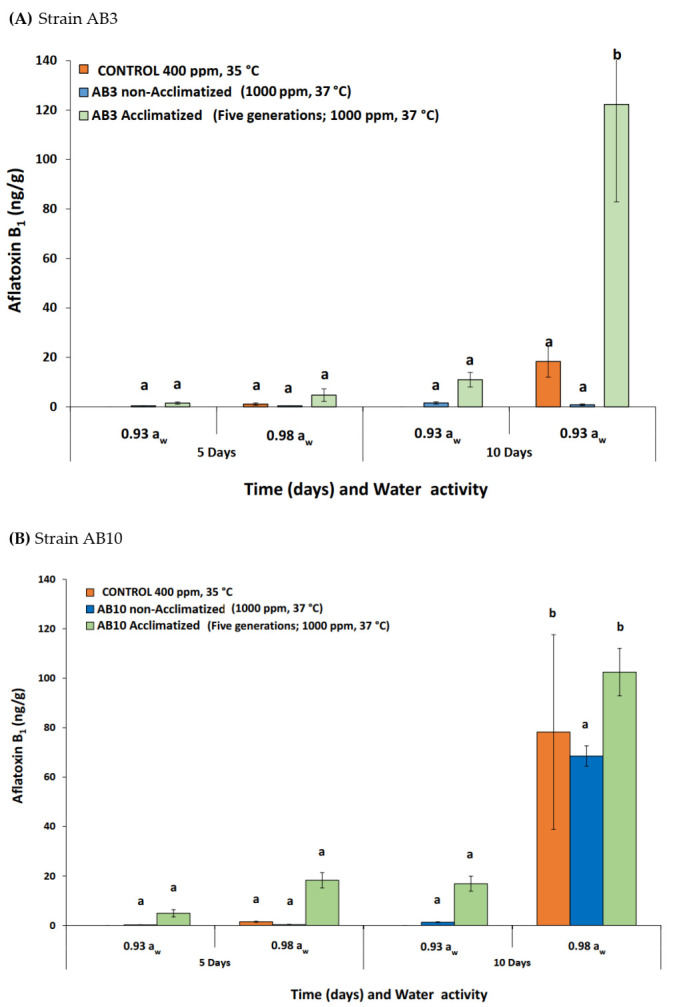
(**A**) Strain AB3 and (**B**) strain AB10: Aflatoxin B_1_ production of non-acclimatized and acclimatized (for five generations) of *Aspergillus flavus* strains on colonised layers of raw pistachio nuts incubated at 35 °C + 400 ppm CO_2_ and 37 °C + 1000 ppm CO_2_. Data are means of triplicates. Different letters indicate significant differences between treatments (*p* < 0.05) by Fisher’s least significant difference (LSD).

Figure 2A,B compares the production of AFB_1_ by the original culture vs. the acclimatized cultures of AB3 and AB10 when exposed to the interacting climate-related abiotic parameters after 5 and 10 days on raw pistachio nuts. Overall, less AFB_1_ contamination of the pistachio nuts occurred after 5 days than after 10 days, especially in the wetter 0.98 a_w_ treatment, regardless of the temperature or CO_2_ exposure treatment. For strain AB3, the production of AFB_1_ was significantly stimulated, especially after 10 days of colonization, when exposed to 37 °C and 1000 ppm CO_2_. However, for the AB10 strain, there was no significant increase when compared to the control, when exposed to existing or future climate-related abiotic conditions (Figure 2B). This points to probable strain variation, even after the acclimatization of this *A. flavus* strain for five generations. In addition, no AFB_1_ was produced in the drier 0.93 a_w_ control treatments (400 ppm CO_2_ + 35 °C) for both strains.

## 4. Discussion

This study suggests that the effect of acclimatization on the colonization of pistachio nuts and perhaps other nuts may need further investigation. The effect of the acclimatization treatment on the two strains differed, although both were isolated from pistachio nuts [8]. The resilience of one strain was increased by acclimatization with faster colonization of the raw pistachio nuts and a clear stimulation of AFB_1_ production. In contrast, for the other strain, there was no difference from the control treatments. It may be that comparisons need to be made of such strains after 5, 10, or perhaps 20 generations in climate-related abiotic conditions in order to determine the real impact of the adaptation and stability of resilience to these factors and what influence this may have on toxin biosynthesis.

The only comparable study was by Vary et al. [10], who used 10 and 20 generations of acclimatization of wheat fungal pathogens. However, they did not examine inter-strain differences. This certainly requires more in-depth research to examine how exposure to climate-related abiotic conditions for different generations affects the expression of the key genes involved in the biosynthesis of AFs. Certainly, changes in the relative expression of some of the biosynthetic genes (*aflD* and *aflR*) in *A. flavus* under climate change-related abiotic factors in pistachio nuts and in maize have been shown to occur [5,6,9]. Indeed, Vary et al. [10] found that *F. graminearum* exposed for 10 generations to elevated CO_2_ had a higher pathogenicity, with more fungal biomass of this species present in the ripening ears of wheat, as well as an increase in visible symptoms. Unfortunately, the effects on deoxynovalenol contamination of the wheat grain were not quantified. The only other study was with *F. verticillioides* and *F. graminearum*, where step changes were made by increasing the temperature slowly for 10 generations. However, this acclimatization was done under existing CO_2_ conditions only. They found little impact on growth or on fumonisins or type B trichothecenes and zearalenone production, respectively [11]. Medina et al. [13] and Perrone et al. [3] pointed out that the three-way interaction between increased temperature, exposure to elevated CO_2_, and drought stress together may be more important for influencing the evolving resilience of such fungal pathogens than single or two-way climate-related abiotic factors.

If we consider climate-related abiotic factor effects without acclimatization, then certainly they suggest significant influences on mycotoxin production in different food-related matrices. Thus, colonization and ochratoxin A (OTA) production by strains of *A. westerdijkiae* were stimulated, while there was practically no effect on strains of *A. carbonarius* [14,15] in coffee-based media and stored coffee beans. Studies of the colonization of maize by *A. flavus* strains under existing and three-way climate-related abiotic factors showed little effect on growth but a significant stimulation of AFB_1_ contamination of maize [7].

In contrast to studies with coffee, strains of *A. carbonarius* from grapes were found to be significantly affected by fluxes in day/night temperature, elevated CO_2_ (400 vs. 1000 ppm), and drought/wet conditions [16]. This study showed an up-regulation of both structural (*AcOTApks*, *AcOTAnrps*, *AcOTAhal, AcOTAp450*, and *AcOTAbZIP*) and regulatory genes of the velvet complex (*laeA*/*veA*/*velB*, “velvet complex”) involved in OTA biosynthesis and phenotypic OTA production.

Other studies with non-xerophilic species such as *Fusarium langsethiae*, which grows under cooler conditions on oats, were also found to respond to three-way climate-related abiotic factors [17]. These showed that the *Tri5* gene expression was reduced in all conditions except at the elevated temperature, 30 °C, when compared to 25 °C, and exposure to 1000 ppm CO_2_ with a 5.3-fold significant increase in expression. Other biosynthetic genes (*Tri6* and *Tri16*) were upregulated in elevated CO_2_ conditions. In stored oats, at 0.98 a_w_, elevated CO_2_ led to a significant increase (73-fold) in the T2/HT-2 toxin, especially at 30 °C [17]. With *F. verticillioides*, Vaughan et al. [18] showed an increase in pathogen biomass during the infection of ripening maize cobs, but no effect on fumonisins production when exposed to 650 ppm CO_2_. However, when drought stress was included, there was a stimulation of fumonisins in ripening maize cobs [19].

In terms of further investigations of acclimatization, perhaps a broader approach needs to be utilized including RNAseq, that may help to better understand the possible role of acclimatization, in relation to changes in the transcriptome and key biosynthetic pathways, for the production of other toxic secondary metabolites by *A. flavus*, including cyclopiazonic acid and related compounds. This would provide a better understanding of the physiological/functional basis for the evolution of resilience in such acclimatized strains. Perhaps acclimatization for at least 10–20 generations in elevated CO_2_ + increased temperature stress is required in order to better understand the evolution of the potential development of tolerance and effects on secondary metabolite production in climate change scenarios.

## 5. Conclusions

This study showed that when *A. flavus* was acclimatized for five generations in elevated CO_2_ on raw pistachio nuts, there were differential strain effects on growth and AFB_1_ production. Colonization of raw pistachio nuts by the original AB3 and B10 strains and the equivalent acclimatized strains were relatively similar, when compared under different climate related abiotic factors of temperature, CO_2_ exposure, and water availability conditions. The effect of climate-related interacting abiotic factors on AFB_1_ production varied with the strain used. For the AB3 strain, there was stimulation of the acclimatized strain when compared to the original strain at 37 °C, 1000 ppm CO_2_ and 0.98 a_w_. However, for the AB10 strain, especially at 0.93 a_w_ after both 5 and 10 days of incubation, there was a stimulation in the AFB_1_ production of the acclimatized strain when exposed to 37 °C and 1000 ppm CO_2_. It may thus be important to consider more strains of this species isolated from pistachio nuts and to acclimatize them for at least 10 generations to identify whether the increase in resilience is consistent and whether toxic secondary metabolite profiles would also be changed when exposed to climate-related interacting abiotic factors. This would provide useful information on the relative risk of increased mycotoxin contamination of this tree nut.

## Data Availability

The data have been deposited with Cranfield University, and are accessible and available via N.M. and A.M., the supervisors.
